# Oral vitamin D supplemental therapy to attain a desired serum 25-hydroxyvitamin D concentration in essential healthcare teams

**DOI:** 10.1186/s13063-022-06944-z

**Published:** 2022-12-16

**Authors:** Banafshe Hosseini, Cécile L. Tremblay, Cristina Longo, Shirin Glochi, John H. White, Caroline Quach, Louis-Georges Ste-Marie, Robert W. Platt, Francine M. Ducharme

**Affiliations:** 1 Clinical Research and Knowledge Transfer Unit On Childhood Asthma, Research Center, Centre Hospitalier Universitaire Sainte-Justine, Montreal, QC Canada; 2 Department of Microbiology, Infectious Disease and Immunology, Centre Hospitalier Universitaire de Montréal, University of Montreal, Quebec, Canada; 3 Department of Pharmacy, University of Montreal, Montreal, QC Canada; 4 Department of Epidemiology, Biostatistics and Occupational Health, McGill University, Montreal, QC Canada; 5 Departments of Physiology and Medicine, McGill University, Montreal, QC Canada; 6 Department of Microbiology, Infectious Diseases and Immunology, Centre Hospitalier Universitaire Sainte-Justine, University of Montreal, Quebec, Canada; 7 Department of Medicine, Centre Hospitalier Universitaire de Montréal, Montreal, QC Canada; 8 Departments of Pediatrics and of Social and Preventive Medicine, University of Montréal, Quebec, Canada

**Keywords:** Vitamin D, Hybrid study, Healthcare workers, Randomized controlled trial

## Abstract

**Background:**

The study objectives were to ascertain the efficacy of vitamin D supplementation in rapidly increasing serum vitamin D and of implementation of a hybrid (virtual and in-person) trial.

**Methods:**

In a randomized triple-blind controlled trial, healthcare workers were allocated to receive an oral bolus of 100,000 IU with 10,000 IU/week of vitamin D_3_ or placebo. The co-primary outcomes were the change from baseline in serum 25-hydroxyvitamin D [(Δ) 25(OH)D] and proportion with vitamin D sufficiency (25(OH)D ≥ 75 nmol/L), at endpoint. Adherence to supplements and procedures as well as adverse event rates were documented.

**Results:**

Thirty-four (19 intervention, 15 control) subjects were randomized, with 28 (41%) virtual visits. After 44.78 ± 11.00 days from baseline, a significant adjusted group difference of 44.2 (34.7, 53.8) nmol/L was observed in the Δ 25(OH)D (95% CI) in favor of supplementation; 77.8% of intervention, and 13.3% of control, patients were vitamin D sufficient (OR:6.11, 95% CI:1.6, 22.9*).* The adherence to intervention was 94.7% in the intervention and 100% in the control groups. Irrespective of visit type, high adherence was observed in sampling procedures and completion of fortnightly online questionnaire. No adverse events attributable to vitamin D were reported.

**Conclusion:**

The vitamin D supplementation rapidly and safely raised 25(OH)D levels to sufficient levels for a biological effect. Similarly high adherence to study procedures was observed with virtual and in-person participation.

**Trial registration:**

This trial was registered at https://clinicaltrials.gov on July 23, 2020 (#NCT04483635).

**Supplementary Information:**

The online version contains supplementary material available at 10.1186/s13063-022-06944-z.

## Background

The global pandemic of severe acute respiratory syndrome coronavirus 2 (SARS-CoV-2) has been regarded as the largest public health problem in recent decades [1]. Concurrent with the development of COVID-19 vaccines, vitamin D supplementation emerged as a potential primary prevention approach to reduce the incidence and severity of SARS-CoV-2 infection. Indeed, a meta-analysis of 27 observational studies showed higher odds of COVID-19-related hospitalization and mortality in subjects with vitamin D deficiency than their counterparts [2]. Several mechanisms support the potential benefits of vitamin D for COVID-19, particularly via its immunomodulatory effects that enhance antiviral immune response and anti-inflammatory properties [3]. A reduced risk of several infections (such as tuberculosis, influenza, and viral upper respiratory tract illnesses) was observed with serum 25-hydroxyvitamin D [25(OH)D] levels between 75 and 80 nmol/L [4, 5]. Vitamin D toxicity is extremely rare, and safety of 10,000 IU/day [6, 7] or 50,000–100,000 IU/week [8] was shown in previous studies. However, the optimal dose of Vitamin D supplementation that can rapidly and safely enhance the serum 25(OH)D to a similar level needs to be determined.

In northern countries with minimal sun exposure, 6–18 weeks of 1000, 2000, or 4000 IU/day are required to raise 25(OH)D levels to 75 or 80 nmol/L [9, 10]. A systematic review of 30 studies showed a rapid increase in 25(OH)D to sufficient levels after single oral bolus doses of ≥ 100,000 IU, with levels peaking after 7 to 30 days [11]. However, boluses alone failed to sustain 25(OD)D ≥ 75 nmol/L beyond 30 days [12, 13] to three months [14–17]. Moreover, a previous meta-analysis showed that compared to daily or weekly vitamin D supplementation, bolus doses might be less effective for the prevention of acute respiratory tract infection [18]. A previous trial in preschoolers with asthma showed that a vitamin D bolus of 100,000 IU followed by 400 IU daily supplements resulted in a rapid rise in total serum 25(OH)D within 10 days and maintained vitamin D sufficiency at 3 months in 100% of the intervention group [19]. A combination of 100,000 IU bolus with daily or weekly supplements would likely achieve a rapid and sustained 25(OH)D serum level as shown in pediatrics [19], which could, in turn, be associated with a rapid protective effect.

The COVID-19 pandemic has interfered with the traditional execution of clinical trials, based on in-person research visits. Yet, virtual visits and monitoring could offer promising advantages such as faster enrolment and increased participant diversity, while reducing unnecessary travel and potential exposure to SARS-CoV-2 [20–22]. However, a virtual trial increases technological demands on participants and raises specific challenges about the feasibility of biological sample collection, and uncertainties about drug and protocol adherence, compared to traditional trials with in-person visits.

Due to the premature termination of our primary prevention trial assessing the impact of vitamin D supplementation on the prevention of COVID-19 in healthcare workers, in this ancillary study, we focused on the impact of a large bolus followed by a weekly dose of vitamin D_3_ on serum vitamin D measured at endpoint. The two co-primary outcomes were the group differences in the: (i) change from baseline in serum 25(OH)D levels and (ii) proportion of subjects achieving vitamin D sufficiency (25(OH)D ≥ 75 nmol/L) at endpoint. In addition, we aimed to explore the adherence challenges of conducting a virtual clinical trial, and thus we compared the study protocol compliance in participants enrolled remotely vs. in-person. Additional exploratory outcomes included adherence to intervention, group difference in the change from baseline in C-reactive protein (CRP) levels as a marker of systemic inflammation, adverse event rate, incidence of laboratory-confirmed COVID-19 infection, and change from baseline in immunoglobulin G (IgG) SARS-CoV2 serology conversion in those who received COVID-19 vaccination during the trial.

## Methods

### Trial design

We initiated a 16-week randomized, parallel-group, triple-blinded, multicentre placebo-controlled trial of vitamin D_3_ (cholecalciferol) supplementation in healthcare workers as a primary prevention of SARS-CoV2 infection. The study was approved by the research ethics board (REB) of the Sainte-Justine University Health Centre (#MP-21–2021-3044), serving as the central Ethics Board as well as the local REB of all participating institutions. Written informed consent was obtained from all participants prior to study participation. This RCT was registered at https://clinicaltrials.gov (# NCT04483635). The study recruitment and follow-up was prematurely terminated due to severe enrolment difficulty as a result of the rapid vaccine rollout in Canada. This study is reported according to recommended standards (Additional file 1).

### Participants

Briefly, healthcare workers aged ≥ 18 and < 70 years, authorized to practice in the Province of Quebec were eligible if they were expected to work in a setting at high risk of contact with COVID-19-infected individuals over the next 16 weeks (i.e., frontline healthcare workers), resided in the greater Montreal area, were covered by the public system for medical services and able to communicate electronically. Participants were excluded if they had: taken greater than 400 IU/day (or 36,000 IU cumulative dose) in the past 3 months or had the intention of taking more than 400 IU/day of vitamin D during the study period; suspected or previously documented COVID-19 infection; a prior COVID-19 vaccination; a history of disorder involving calcium or vitamin D metabolism; active cancer; oral medication interfering with vitamin D metabolism; anticipated prolonged absence from work or difficult follow-up; or enrolled in a concurrent interventional trial.

### Intervention and randomization

Participants were randomly assigned to the receiving 100,000 IU oral bolus at randomization followed by a weekly dose of 10,000 IU of vitamin D_3_, or placebo bolus and weekly supplement of placebo until the study endpoint. Randomization was implemented using a computer-generated random list stratified by health administrative regions. To maintain blinding, the intervention and placebo dose were identical in color, appearance, volume, taste, and packaging. All research personnel, physicians, nurses, and participants were blinded to group allocation. The effectiveness of blinding was assessed at endpoint by independently asking each participant and associated research coordinator to guess the participant’s study group assignment (intervention, control, unable to guess): it was reported as a percentage of correct guess [23].

### Study outcomes and measurements

Our original primary outcome was the incidence of laboratory-confirmed SARS-CoV2 infection; however, after premature trial cessation due to difficulty in enrollment in the context of mass COVID-19 vaccination, the co-primary outcomes were modified post hoc to the overall change (Δ) from baseline in total serum 25(OH)D and proportion of participants with serum 25(OH)D ≥ 75 nmol/L at endpoint, assessed by DiaSorin Liaison assay. Exploratory outcomes included between group differences in: (i) adherence to study supplements (verified via pill-count method); (ii) adherence to study intervention and procedures (i.e., sample collection, biweekly electronic questionnaire); (iii) change from baseline in serum CRP level (measured by enzyme-linked immunoassay (ELISA) using a commercial CRP ELISA kit) as marker of systemic inflammation, (iv) difference in proportion of subjects with serum CRP levels > 5 mg/L (normal threshold in our laboratory) at the endpoint; (v) adverse events (participant-reported outcome); (vi) incidence of laboratory confirmed SARS-CoV2 infection (nasopharyngeal or salivary samples clinically for screening or diagnostic purposes throughout the study or obtained at endpoint, analyzed by RT-qPCR nucleic acid amplification test, or positive IgG SARS-CoV2 antibody at endpoint (i.e., ≥ 15 UA on the anti-S SARS-CoV-2 IgG Diasorin on Liaison XL platform in unvaccinated individuals or ≥ 1.40 index (S/C) on the anti-N SARS-CoV-2 IgG on ARCHITECT platform in individuals vaccinated after randomization); (vii) in the subgroup of subjects vaccinated after randomization, the antibody response to COVID-19 vaccine, measured by change from baseline in the anti-S SARS-CoV-2 IgG Diasorin measured on Liaison XL platform.

### Procedures

**Fig. 1:**
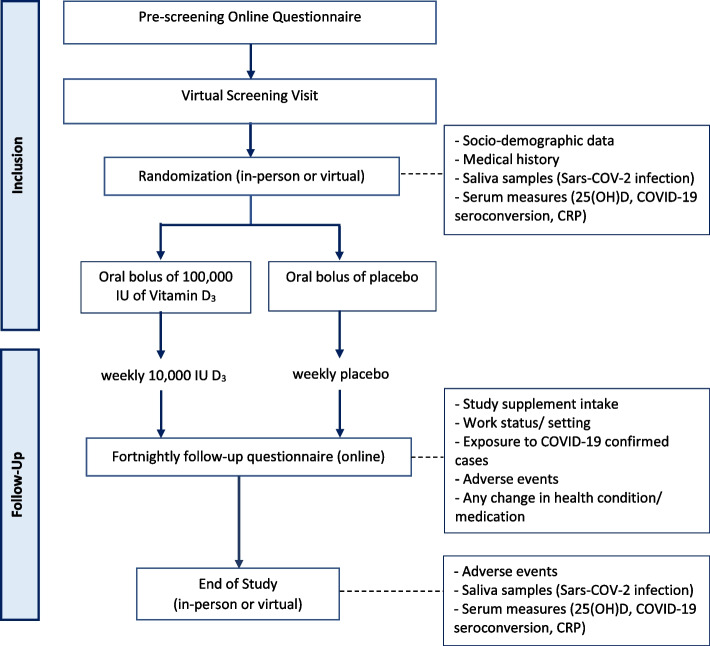
Trial flow chart. Interested individuals were invited to complete a pre-screening online questionnaire; if potentially eligible, a brief written summary of the study intervention and procedures along with the study consent form was provided online; potential eligible subjects were invited to self-schedule themselves for a virtual screening visit by Zoom teleconferencing, to confirm eligibility with a research coordinator, and confirmed eligible participants were offered an in-person or remote randomization to be allocated to one of two groups (intervention or placebo). All required materials as well as study supplements were shipped to participants prior to the remote visit. Participants were asked to complete the fortnightly follow-up questionnaire throughout the study

Eligible and consenting participants were offered a randomization visit, either in-person or virtual (Fig. 1). Video demonstration of key procedures (e.g., home blood collection) allowed participants to choose their preferred type of randomization and end-of-study visits (in-person vs. virtual). For those selecting the remote approach, study materials and supplements were shipped by courier to participants prior to the virtual visit and participants were asked to ship collected samples to the coordinating center. Samples included saliva (for qPRC, with/without genetic sampling) and venous or capillary blood; capillary collection was achieved with the TASSO OnDemand SST device (Tasso Ink, Seattle, WA, United States), aiming for a volume of 490uL and 570uL for the randomization and end-of-study visits, respectively. After randomization, participants received electronic notification via either e-mail or text messages: (i) weekly, to remind them to take their weekly study supplement and start reporting symptoms, if any, on the electronic daily diary, and (ii) fortnightly, to complete a brief online health questionnaire. If the health questionnaire was not submitted within a few days, the research coordinator contacted patients to ask or assist them in completing the questionnaire; if not completed within 7 days, a second follow-up was made. Research coordinators were available to participants by phone and email throughout the study. Remote or in-person end-of-study visits were offered.

### Statistical methods

An intention-to-treat (ITT) analysis was carried out with all randomized participants included in the analysis. Continuous variables were reported as mean with standard deviation (SD) and the median with quartiles, respectively. Categorical variables were presented as proportions. Adjusted mean changes in serum levels of 25(OH)D and CRP were computed using the analysis of covariance, with values reported with 95% confidence interval (CI). Potential covariates for the main outcome (Δ25(OH)D) included age, sex, baseline 25OHD, skin color (types 1–6) [24], weight status (non-overweight versus overweight/obese (body mass index (BMI) ≥ 25 kg/m^2^)), comorbidity (yes/no) and the number of weekly doses of Study supplement taken throughout the study. Candidate covariates for the change in CRP were age, sex, baseline CRP, baseline 25(OH)D, weight status, and comorbidity status. The Chi-square or Fisher exact test was used to compare group differences in proportions (e.g., vitamin D sufficiency). Geometric means served to assess the rise in IgG titers following vaccination. All tests were 2-sided and *P* value of less than 0.05 was considered statistically significant. Statistical analysis was performed with SPSS (version 26.9, Chicago, IL).

## Results

**Fig. 2:**
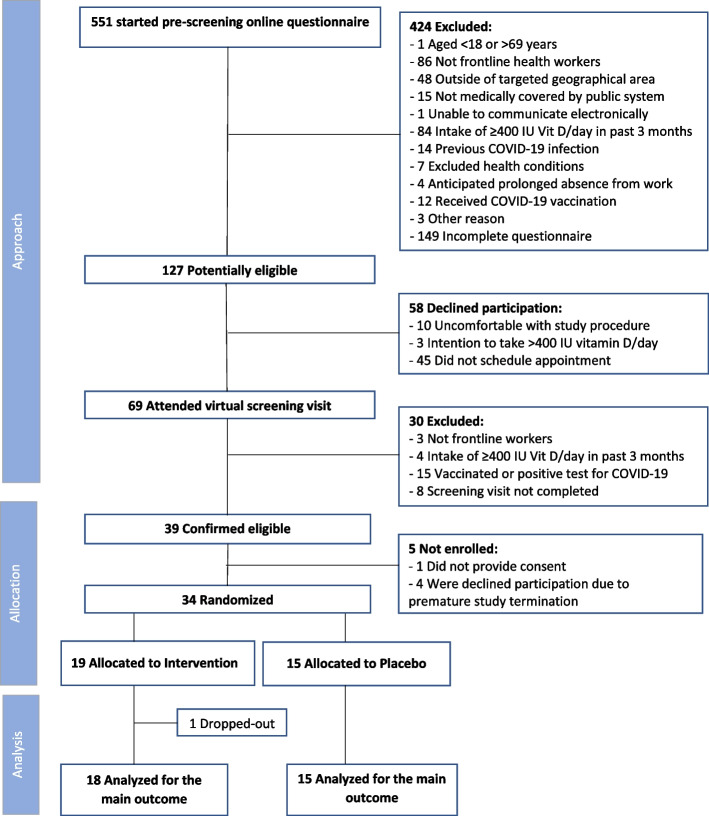
Participant selection. The flow of participants from screening to analysis

From February 8 to May 4, 2021, 551 subjects initiated the online pre-screening questionnaire. Of these, 424 (77%) were not eligible, primarily because they were not frontline workers (20%) or had exceeded the maximum intake of vitamin D in the preceding three months (20%); 149 (35%) did not answer all pre-screening questions resulting in 127 potentially eligible participants; 58 (46%) declined participation, prior to the virtual screening visit. Of 69 individuals attending the virtual screening visit, 30 (43%) were excluded, primarily due to a recent COVID-19 infection or vaccination with 39 subjects confirmed eligible; one did not provide consent and four declined participation due to premature study cessation. Thirty-four (19 intervention; 15 control) subjects were randomized. With one intervention patient dropping out due to personal reasons, 33 participants were included in the analysis of the co-primary outcomes (Fig. 2).

Most participants were female (94%), Caucasian (70.6%), non-smokers (67.6%), with a mean age of 39.5 years; 88% were not taking any vitamin D supplements (Table 1). The majority (91.1%) of participants displayed a baseline serum 25(OH)D level < 75 nmol/L (Table 1). Baseline characteristics were similar between the two groups, with a slightly greater portion of subjects with darker skin types (4–6 on Fitzpatrick scale [24]), normal bodyweight (BMI ≤ 25 kg/m^2^), and fewer baseline comorbidities in the intervention versus the control group.

**Table 1:** Baseline characteristics of intention-to-treat participants

Characteristics	Intervention (*N* = 19)	Control (*N* = 15)
**Demographics**		
**Age (years) *–**** mean* ± *SD*	39.32 ± 11.59	39.6 ± 8.75
**Female** *– n (%)*	19 (100.0%)	13 (86.6%)
**Body mass index (kg/m**^**2**^**) *–**** median (Q1, Q3)*	23.60 (20.80, 29.30)	23.90 (20.60, 27.60)
**Weight status**^**1**^*– n (%)*		
Non-overweight/obese	12 (63.2%)	8 (53.3%)
Overweight/obese	7 (36.8%)	7 (46.7%)
**Ethnicity** *– n (%)*		
North American origins (non-Aboriginal)	8 (42.1%)	7 (46.7%)
European origins	4 (21.1%)	5 (33.3%)
Caribbean and Pacific Islands origins	2 (10.5%)	2 (13.3%)
African origins	4 (21.1%)	1 (6.7%)
Asian origins	1 (5.3%)	0 (0.0%)
**Skin color**^**2**^*– n (%)*		
1–3	14 (73.7%)	13 (86.7%)
4–6	5 (26.3%)	2 (13.3%)
**Smoking status** *– n (%)*		
Never smoker	12 (63.2%)	11 (73.3%)
Former smoker	6 (31.5%)	3 (20.0%)
Passive smoker	1 (5.3%)	0 (0.0%)
Active smoker	0 (0.0%)	1 (6.7%)
**Work title** *– n (%)*		
Nurse/nursing assistant	10 (52.6%)	5 (33.4%)
Technician	0 (0.0%)	3 (20.0%)
Other healthcare professionals	9 (47.4%)	7(46.7%)
**Comorbidity** *– n (%)*		
Overall	7 (36.8%)	9 (60.0%)
Chronic hematologic diseases	0 (0.0%)	1 (6.7%)
General disorders	2 (10.5%)	0 (0.0%)
Musculoskeletal and connective tissue disorders	1 (5.3%)	1 (6.7%)
Infections and infestations	1 (5.3%)	2 (13.3%)
Gastrointestinal disorders	4 (21.1%)	1 (6.7%)
Nervous system disorders	1 (5.3%)	0 (0.0%)
Endocrine disorders	2 (10.5%)	1 (6.7%)
Immune system disorders	0 (0.0%)	3 (20.0%)
**Vitamin D supplementation (IU/day)**^**3**^*– n (%)*		
None	16 (84.2%)	14 (93.3%)
≤ 400 IU	3 (15.8%)	1 (6.7%)
**Baseline serum laboratory values**		
**C-reactive protein (mg/L) *–**** median (Q1, Q3)*	1.00 (0.37, 2.15)	0.80 (0.40, 4.50)
**CRP ≥ 5 mg/dL***– n (%)*	2 (10.5%)	3 (20.0%)
**25-hydroxy vitamin D (nmol/L) –** *mean* ± *SD*	48.65 ± 26.19	48.02 ± 15.16
**25-hydroxy vitamin D status –** *n (%)*		
< 25 nmol/L	1 (5.3%)	1 (6.7%)
25–49.99 nmol/L	12 (63.2%)	7 (46.7%)
50–75 nmol/L	4 (21.1%)	6 (40.0%)
≥ 75 nmol/L	2 (10.5%)	1 (6.7%)

^1^Weight status calculated based on body mass index (BMI) values. Non-overweight: BMI < 25 kg/m^2^; overweight or obese: BMI ≥ 25 kg/m^2^

^2^Ascertained by the 6-point Fitzpatrick’s sun-reactive skin type classification from (1) very light skin to (6) dark skin [24]

The average treatment period was comparable between the two groups (intervention: 43.05 ± 9.01 vs. Control: 46.88 ± 13.01 days) with a range of 27 to 70 days. Approximatively, 40% of visits were conducted remotely, including 14 (10 intervention: 4 control) randomization, and 14 (9 intervention: 5 control) end-of-study visits. After randomization, 5 and 6 subjects in the intervention and control groups, respectively, received their first dose of the COVID-19 vaccine (Pfizer-BioNTech COVID-19 mRNA). There was no evidence of unblinding among participants or research coordinators at the end of the study with 30.3% of participants (Intervention: 22% vs. Control: 40%, *P* = 0.539) correctly guessing the assigned cohort arm. Research coordinators correctly guessed the intervention assignment in only 9% of the participants (11.1% vs. 6.7%, *P* = 0.859).

**Fig. 3:**
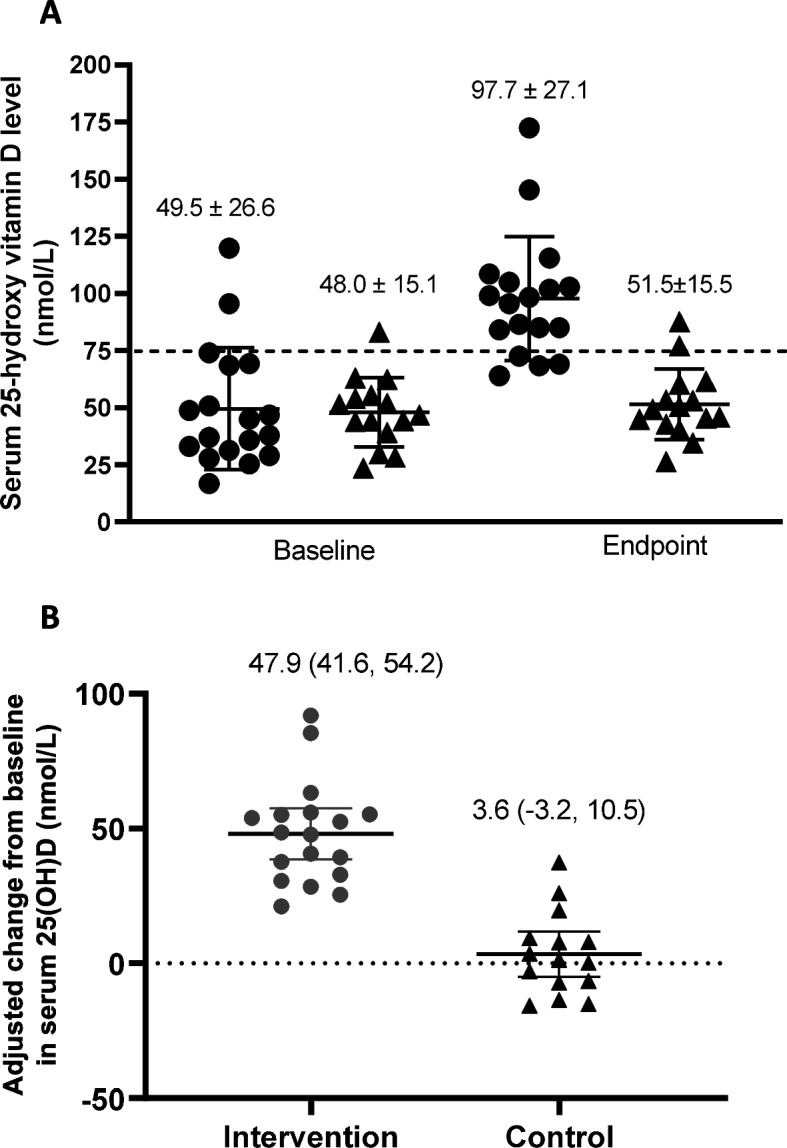
Baseline and endpoint crude 25-hydroxyvitamin D [25(OH)D] levels **A**; and adjusted change from baseline in serum 25(OH)D levels **B** in intervention (circle) and control (triangle) group. Values are adjusted for skin color, baseline weight status, and comorbidity status. Bars and values printed on top of each timepoint/group represent the mean ± SD 25(OH)D level in **A** and mean (95% CI) 25(OH)D level in **B**. Individual participant values are represented by dots. The dotted lines depict 75 nmol/L in **A** and change in 25(OH)D level, in **B**

At endpoint, the intervention group reached a mean ± SD 25(OH)D level of 97.7 ± 27.1 nmol/L compared to 51.5 ± 15.5 nmol/L in the control group (Fig. 3A). There was a 45.37 (95% CI: 35.7, 55.03) greater increase from baseline in serum 25(OH)D in the intervention than the control group, after adjusting for skin color, weight status, comorbidity status, and the number of weekly doses of study supplement received (Fig. 3B). In other words, 78% (*N* = 14) participants in the intervention group became vitamin D sufficient (≥ 75 nmol/L) compared to 13.3% (*N* = 2) subjects in the control group.

The observed adherence to the study bolus administered at randomization was 100% in both groups; adherence to weekly supplement was 94.7% in the intervention, (one drop-out at week 2 was assumed to be non-adherent) and 100% in the control, groups. As for visit procedures, a sufficient amount of blood was collected from venous or capillary self-collection. Of note, most patients needed two Tasso SST devices to collect a sufficient amount of blood at randomization and at endpoint (Table 2). All saliva samples collected by participants throughout the study were acceptable for qPRC analysis.

**Table 2:** Analysis of remote capillary blood collection by visit

Variable	**Randomization (*n* = 14)**	**End of study (*n* = 14)**
**Target blood volume (μL)**	490	570
**# Tasso devices needed to collect sufficient blood – ***n (%)*		
1	2 (14.3%)	0 (0.0%)
2	10 (71.4%)	9 (64.3%)
3	2 (14.3%)	5 (35.7%)
**Blood volume collected per Tasso (μL) –** *mean* ± *SD collected*	229.4 ± 95.7	233.7 ± 67.1

Regarding electronic monitoring, nearly half of the participants (44%) had delayed completion of their first questionnaire and over 10% had delayed completion of the second questionnaire by more than a few days, prompting a contact by the research assistant (Table S1). Other than time constraints, two problems were rapidly identified: (1) the first questionnaire link was not sent in a timely fashion to the virtually enrolled participants due to a programming error and (2) some participants reopened the link to a previously completed questionnaire instead of the latest link. Both issues were rapidly resolved by the CRF programmer or by additional training of the participants, with minimum need of additional prompting by the research coordinator, with over 90% spontaneously timely completion thereafter.

There was no significant group difference in change from baseline in CRP levels in 29 participants contributing data to both baseline and end of study measurements (adjusted mean difference (95% CI): 1.50 mg/L (− 1.85, 4.85) after adjusting for baseline CRP, weight status, and comorbidity status. Yet, significantly fewer subjects displayed elevated serum CRP levels (≥ 5 mg/L) at endpoint in the intervention, compared to the control, group (intervention: 5.5% vs. control: 23.0%) (Figure S1).

Two subjects in the control group (both unvaccinated) and none in the intervention became SARS-CoV2 infected during the study period. Of the 11 subjects vaccinated after randomization, only 6 (2 intervention; 4 control) subjects were sampled 14 days or more after immunization, providing insufficient power to explore any group differences in the rise in IgG.

Overall, eight adverse health events were reported in 8 (2 intervention; 6 control) participants (Table S2). All were deemed minor and none was attributed to vitamin D toxicity.

## Discussion

In the present study, one bolus of 100,000 IU vitamin D_3_ followed by a weekly dose of 10,000 IU vitamin D_3_ rapidly and significantly increased serum 25(OH)D by an average of 48 nmol/L to an adjusted mean of almost 100 nmol/L, which is, in the range where protective immunomodulatory effects have previously been observed [4, 5]. This intervention permitted 78% of participants in the intervention to achieve vitamin D sufficiency (> 75 nmol/L) at endpoint, compared to fewer than 15% of controls, within 27 to 70 days.

Several studies have evaluated the dosage of vitamin D supplements required to safely attain and maintain the optimal serum 25(OD)D level in the general population [16, 19, 25]. Supplementation of 3000 to 5000 IU/day for 6 months can raise 25(OH)D levels to above 75 nmol/L [9], depending on baseline levels. Conversely, in a systematic review of oral vitamin D supplementation, a single dose of 100,000 IU raised serum 25(OH)D concentrations to above 75 nmol/L; however, it only persisted for 5 to 6 weeks when it was < 50 nmol/L and until 3 months if the baseline was > 50 nmol/L [11]. In two pediatric trials, 100,000 IU bolus combined with daily supplement at age-specific recommended doses of 400 IU achieved a rapid (10 days) and sustained (3 months) serum 25(OH)D above 75 nmol/L in all children; but neither boluses alone nor daily supplementation alone was sufficient to lead to a rapid and sustained level about 75 nmol/L [16, 19]. Our findings are in line with the observations from the literature and confirm the values of combining a bolus with regular dosing to achieve rapid and sustained vitamin D sufficiency.

Due to the trial’s premature termination, we were unable to explore the effects of vitamin D supplementation on COVID-19 infection risk. While numerous studies explored the potential impact of vitamin D in patients hospitalized with COVID-19, studies pointing to the primary prevention remain limited to few observational studies [26–28], which provided conflicting results. There are few trials registered at Clinicaltrials.gov addressing the effects of vitamin D supplementation on the risk of COVID-19 infection and completion of these ongoing trials is needed to expand our understanding of the effects of vitamin D supplementation on preventing COVID-19.

As one of the potential mechanisms of action of vitamin D in COVID-19 is via its potential anti-inflammatory properties, we explored the impact of supplementation on serum CRP. Indeed, vitamin D supplementation could suppress the nuclear factor kappa B (NF-κB) pathway, which in turn may reduce systemic inflammation and production of CRP [29]. While our observed group difference of 1.50 mg/L in change from baseline in serum CRP did not reach statistical significance, fewer subjects had elevated CRP (> 5 mg/L) in the intervention, than in the control, group at endpoint, after adjusting for baseline levels and baseline group imbalances. A previous meta-analysis of 10 trials involving a total of 924 participants indicated that daily vitamin D supplementation (ranging from 400 to 7143 IU for 8 to 48 weeks) significantly decreased the circulating CRP level by 1.08 mg/L (95% CI, − 2.13, − 0.03), with evidence of significant heterogeneity [29]. Additional well-designed RCTs are warranted to further investigate the role of vitamin D supplementation in systemic inflammation.

Adherence to the intervention was high in both groups probably in part due to weekly rather than daily administration regimen, coupled with an electronic reminder, as both techniques have previously been shown to improve adherence [30–33]. Furthermore, with clear written instructions, step-by-step video guide, as well as virtual supervision during sample collection, our participants demonstrated that they could collect their samples and shipped it with appropriate packaging, with all samples received in acceptable condition. Although slightly more than half of the participants completed the first few fortnightly health questionnaires within the requested timeframe, rapid follow-up of delayed completion identified errors that were resolved quickly, which resulted in over 90% timely completion of study questionnaires throughout the trial. Thus, adherence to the protocol should be closely monitored, which would allow rapid identification and correction of relevant errors. Furthermore, our study population was healthcare workers, given this cohort's likely high degree of compliance to study intervention and protocol, the observed adherence in our study is almost certainly higher than would be expected in the general public. Moreover, we cannot rule out the possibility that the adherence could have reduced with a longer study duration. The literature reports mixed results regarding the implementation of virtual trials. Similar to our findings, some studies showed overall success in adherence to intervention and study procedures [34–36], while others reported higher dropout rates [37, 38]. Conducting a hybrid trial during the COVID-19 pandemic has allowed us to conduct research activities, even during lockdown and curfew periods. With close monitoring and supervision, it could be a promising alternative for in-person visits even after the pandemic.

No adverse health events attributable to vitamin D toxicity were reported throughout the study, which is in line with previous research that reported daily doses of vitamin D up to 10,000 IU were safe in adult participants [4, 7, 39].

The strengths of this trial include the randomized controlled design and high retention rate (97%). We acknowledge several limitations, including the inconsistent study duration among the participants, varying between 27 and 70 days, due to premature study termination. Nonetheless, the duration was adequate to detect important between groups differences in serum 25(OH)D levels. Although data regarding dietary vitamin D intake or sunlight exposure was not collected throughout the study; the serum 25OHD level remained stable in the control study, suggesting no meaningful impact of these factors on study findings.

Our findings can be generalized to both Canadian adults and healthcare workers. Indeed, our observations are consistent with a previous study reporting vitamin D insufficiency in winter and early spring in over 70% of Canadians, with only one third supplementing their diet, usually with < 400 IU vitamin D/ day [40]. Our findings are also concordant with a previous systematic literature review showing that above 92% of nurses and practicing physicians had serum vitamin D levels < 75 nmol/L [41].

## Conclusions

In summary, our study indicates that the administration of an oral bolus of 100,000 IU vitamin D_3_ followed by a weekly dose of 10,000 IU vitamin D_3_ was an effective approach to rapidly and safety raise serum 25(OH)D in adult healthcare workers. Our study also provided proof of concept that implementing a hybrid (virtual and in-person) trial could maintain high adherence to the study intervention and procedures. Lessons learned from this study can be used in methods development and the design of future hybrid trials.

## Supplementary Information

**Additional file 1. **CONSORT 2010 Checklist.

**Additional file 2: Table S1. **Adherence to Study Follow up Questionnaire by Randomization visit format. Table S2- Adverse Health Events reported throughout the study. Figure S1- Box plot of unadjusted C-reactive protein levels at baseline and endpoint.

## Data Availability

The datasets used and/or analyzed during the current study are available from the corresponding author on reasonable request.

## Abbreviations

25(OH)D: 25-Hydroxyvitamin D
RCT: Randomized controlled trial
SARS-CoV-2: Severe acute respiratory syndrome coronavirus 2
CRP: C-reactive protein
IgG: Immunoglobulin G
REB: Research ethics board
ELISA: Enzyme-linked immunoassay
ITT: Intention-to-treat
SD: Standard deviation
BMI: Body mass index
NF-κB: Nuclear factor kappa B
