# Supplementary material for: Oral vitamin D supplemental therapy to attain a desired serum 25-hydroxyvitamin D concentration in essential healthcare teams
Source: Trials. 2022 Dec 16;23:1019. doi: 10.1186/s13063-022-06944-z (PMC9756469; doi:10.1186/s13063-022-06944-z)
Supplement: Supplementary file 2 — Additional file 2: Table S1. Adherence to Study Follow up Questionnaire by Randomization visit format. Table S2- Adverse Health Events reported throughout the study. Figure S1- Box plot of unadjusted C-reactive protein levels at baseline and endpoint. [file 13063_2022_6944_MOESM2_ESM.pdf]

Supplemental Document

Table of Contents

Table S1- Adherence to Study Follow up Questionnaire by Randomization visit format 2

Table S2- Adverse Health Events reported throughout the study.. 3

Figure S1- Box plot of unadjusted C-reactive protein levels at baseline and endpoint................................ 4

Table S1- Adherence to Study Follow up Questionnaire by Randomization visit format

**Fortnightly questionnaire completed-** *n (%)*		**In-person visit**	**Virtual Visit**
Week 2	Number of applicable participants	20	14
	Spontaneous completion	12 (60.0%)	6 (42.9%)
	Upon first follow-up by Research personnel	7 (35.0%)	7 (50.0%)
	Upon second follow-up by Research personnel	1 (5.0%)	1 (7.1%)
Week 4	Number of applicable participants	19	13
	Spontaneous completion	17 (89.5%)	10 (76.9%)
	Upon first follow-up by Research personnel	2 (10.5%)	1 (7.7%)
	Upon second follow-up by Research personnel	0 (0.0%)	2 (15.4%)
Week 6	Number of applicable participants	11	7
	Spontaneous completion	10 (90.9%)	7 (100.0%)
	Upon first follow-up by Research personnel	0 (0.0%)	0 (0.0%)
	Upon second follow-up by Research personnel	1 (9.1%)	0 (0.0%)
Week 8	Number of applicable participants	4	1
	Spontaneous completion	3 (75.0%)	0 (0.0%)
	Upon first follow-up by Research personnel	1 (25.0%)	1 (100.0%)
	Upon second follow-up by Research personnel	0 (0.0%)	0 (0.0%)

[1] When questionnaire was not submitted within a few days after receiving the invitation link

[2] When questionnaire was not submitted within 7 days after receiving the invitation link

Table S2- Adverse Health Events reported throughout the study				
All adverse health events	**Intervention (N=19)**		**Control (N=15)**	
	Events n	Patients n (%)	Events n	Patients n (%)
Blood and lymphatic system disorders	1	1 (5.2%)	1	1 (6.7%)
General disorders	0	0 (0.0%)	1	1 (6.7%)
Metabolism and nutrition disorders	0	0 (0.0%)	1	1 (6.7%)
Musculoskeletal and connective tissue disorders	1	1 (5.2%)	1	1 (6.7%)
Respiratory disorders	0	0 (0.0%)	2	2 (13.4%)

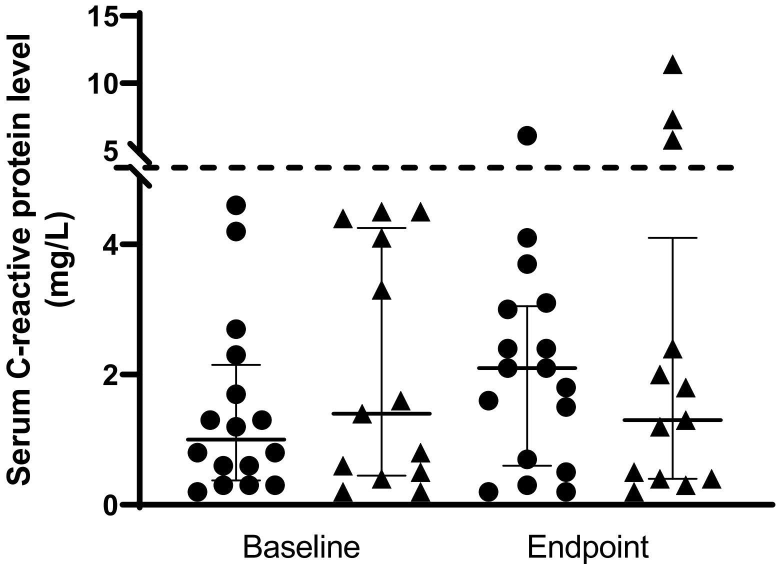

Figure S1- Box plot of unadjusted C-reactive protein levels at baseline and endpoint in intervention (full circle) and control (full triangle) group. The middle bar represent the median, with 25% and 75% percentiles. The dotted line depicts 5mg/L (cut-off value for normal range in adults)
